# Impact of Astaxanthin on Diabetes Pathogenesis and Chronic Complications

**DOI:** 10.3390/md18070357

**Published:** 2020-07-09

**Authors:** Rebecca Landon, Virginie Gueguen, Hervé Petite, Didier Letourneur, Graciela Pavon-Djavid, Fani Anagnostou

**Affiliations:** 1 CNRS UMR7052-INSERM U1271, Laboratory of Osteoarticular Biology, Bioengineering and Bioimaging, Paris Diderot University, 10 Avenue de Verdun, 75010 Paris, France; rebecca.landon@inserm.fr (R.L.); herve.petite@univ-paris-diderot.fr (H.P.); 2 INSERM U1148, Laboratory for Vascular Translational Science, Cardiovascular Bioengineering, Sorbonne University Paris Nord, 99 Avenue Jean-Baptiste Clément, 93430 Villetaneuse, France; virginie.gueguen@sorbonne-paris-nord.fr (V.G.); didier.letourneur@inserm.fr (D.L.); graciela.pavon@univ-paris13.fr (G.P.-D.); 3 Service of Odontology, Hôpital Pitié-Salpêtrière APHP, U.F.R. of Odontology, Denis-Diderot University, 47-83 Boulevard de l’Hôpital, 75013 Paris, France

**Keywords:** astaxanthin, diabetes mellitus, oxidative stress, hyperglycemia, diabetes complication, diabetic retinopathy, diabetic neuropathy, diabetic nephropathy, insulin resistance, antioxidant, ROS

## Abstract

Oxidative stress (OS) plays a pivotal role in diabetes mellitus (DM) onset, progression, and chronic complications. Hyperglycemia-induced reactive oxygen species (ROS) have been shown to reduce insulin secretion from pancreatic β-cells, to impair insulin sensitivity and signaling in insulin-responsive tissues, and to alter endothelial cells function in both type 1 and type 2 DM. As a powerful antioxidant without side effects, astaxanthin (ASX), a xanthophyll carotenoid, has been suggested to contribute to the prevention and treatment of DM-associated pathologies. ASX reduces inflammation, OS, and apoptosis by regulating different OS pathways though the exact mechanism remains elusive. Based on several studies conducted on type 1 and type 2 DM animal models, orally or parenterally administrated ASX improves insulin resistance and insulin secretion; reduces hyperglycemia; and exerts protective effects against retinopathy, nephropathy, and neuropathy. However, more experimental support is needed to define conditions for its use. Moreover, its efficacy in diabetic patients is poorly explored. In the present review, we aimed to identify the up-to-date biological effects and underlying mechanisms of ASX on the ROS-induced DM-associated metabolic disorders and subsequent complications. The development of an in-depth research to better understand the biological mechanisms involved and to identify the most effective ASX dosage and route of administration is deemed necessary.

## 1. Introduction

Diabetes mellitus (DM), the most common metabolic disease, has become a major health concern with increasing prevalence worldwide. Currently, 463 million people (9.3% of adults aged 20–79 years) are living with diabetes around the world. A further 1.1 million children and adolescents under the age of 20 live with type 1 diabetes (T1DM) [1]. This number is projected to increase to 578 million by 2030. The financial burden of DM is also staggering. The cost of the total diabetes-related healthcare is estimated to be around $760 billion and has been projected to reach up to $825 billion in the next 10 years. DM is a chronic disease that is recognized by hyperglycemia (HG) resulting from impaired insulin secretion, inappropriate insulin action, or both. The insulin deficit leads to high levels of blood glucose (HG), which, if not tightly controlled, leads to disabling and life-threatening health complications including cardiovascular diseases, retinopathy, neuropathy, nephropathy, and prolonged/incomplete wound healing [1]. Moreover, DM increases bone fragility and impaired bone healing [2,3]. The main categories of DM are type 1 (T1DM), type 2 (T2DM), and gestational DM. From these three types, T2DM is the most common type, accounting for around 90% of all diabetes worldwide.

The development of DM and its complications are known to be associated with oxidative stress (OS) and low-grade chronic inflammation [4]. OS, an imbalance between cellular oxidant and antioxidant systems, results from the overproduction of free radicals and associated reactive oxygen species (ROS). HG upregulates the markers of chronic inflammation and contributes to increased ROS generation, which ultimately involves DM complications including vascular dysfunction [5]. Moreover, an increased level of ROS reduces insulin secretion and impairs insulin sensitivity and signaling in insulin-responsive tissues [5]. Proper treatment of HG and inhibition of ROS overproduction is, therefore, crucial for delaying the DM onset and progression as well as for preventing its subsequent complications.

Recent advances in biological properties of antioxidants such as carotenoids have suggested that these compounds are not only able to prevent but also able to ameliorate diabetes and its subsequent complications [6]. In particular, astaxanthin (ASX) (3,3′-dihydroxy-β, β′-carotene-4,4′-dione), a xanthophyll carotenoid [7], has been reported to exhibit multiple biological activities including modulation of OS and inflammation through free radical quenching [8] and activation of endogenous antioxidant systems via modulation of gene expression [9,10] and has numerous advantages compared to some other carotenoids. Due to its unique structure, ASX is incorporated in the lipid bilayer of cellular membranes, without damaging it, and prevents lipid-based oxidation [11]. Moreover, its bioactivity is by far higher than other carotenoids such as α-carotene, β-carotene [12], and α-tocopherol [13,14]. It has therefore attracted considerable interest because of its potential pharmacological effects including antidiabetic, anti-inflammatory, and antioxidant activities properties and anti-inflammatory; antidiabetic; as well as its neuro-, nephro-, and retinoprotective and cardiovascular effects [6].

Given the crucial role of OS and inflammation in the pathogenesis and subsequent complications of DM, in this review, we aimed to analyze the biological effects and the underlying mechanisms of ASX on the prevention and the treatment of DM-associated pathologies.

## 2. Pathophysiology of Diabetes-Related Metabolic Disorders and Complications Focus on the Hyperglycemia–Oxidative Stress Interactions

T1DM and T2DM are complex conditions characterized by elevated blood glucose levels due to impaired insulin production and/or to a reduced insulin sensitivity and function. T1DM is caused by the autoimmune-mediated destruction of Langerhans-islet-β-cells and is mainly associated with failure in insulin production, while T2DM is associated with chronic abnormalities of carbohydrate and fat metabolisms in which triggering various pathways induces impaired insulin secretion from the pancreatic β-cells, insulin resistance, reduced glucose utilization in peripheral tissues, and increased hepatic glucose production [15]. In both T1DM and T2DM, chronic HG leads to macro- and microvascular complications [16]. Today, it is admitted that OS and ROS are involved in the pathogenesis of impaired insulin secretion from the pancreatic β-cells, insulin resistance development, and endothelial dysfunction in both T1DM and T2DM [15,16,17] and are deeply altered by oxidation DNA, RNA, protein, and lipid molecules which can be used as possible markers for disease progression [16].

In diabetes-related pathologies, ROSs play a central role in the interactions involving metabolic control, OS, and inflammation [4,18]. ROSs are a family of species including molecular oxygen and its derivatives: superoxide anion (O^2−^), nitric oxide (NO), peroxynitrite (ONOO^−^), hydroxyl (HO), hydrogen peroxide (H_2_O_2_), hypochlorous acid (HOCl), and lipid radicals. Out of these molecules, superoxide has been the initial oxygen free radical, which is then converted into other more reactive species that can impair cell function. They are produced in aerobic metabolism at very low levels and play a crucial role in cell signaling homeostasis [19]. ROSs are mainly produced in the mitochondrial electron transport chain (ETC), endoplasmic reticulum (ER), peroxisomes, and microsomes [4]. Overproduction and/or insufficient removal of excessive ROS by endogenous antioxidant systems result in damage to cellular proteins, membrane lipids, and nucleic acids, thereby leading to OS. Besides antioxidant components such as superoxide dismutase (SOD) and glutathione (GSH), different cellular compartments (nucleus, cell membrane, and mitochondria) contribute differently to the ROS balance [18].

In HG conditions and/or fatty acid oxidation, therefore, ROS overproduction causes cellular dysfunction, apoptosis, and inflammation in several insulin targets including the pancreas, liver, kidney, nerves, and vasculature [17,18].

For instance, in T1DM, the generated ROS under metabolic or autoimmune stress in the cells of the pancreatic microenvironment may enhance damage of pancreatic β-cells, thereby reducing insulin secretion [17]. Regarding T2DM, HG-induced ROS production is considered a major contributor to damage and disease progression. HG leads to glucose autoxidation, alters mitochondrial dynamics, and enhances ROS production. The induced OS reduces insulin secretion and impairs insulin signaling in target tissues [20]. ROS overproduction is also the underlying contributor to the development of vascular complications of DM [18]. Specifically, in the HG-induced vascular damage, several mechanisms are involved: (1) increased flow of glucose mediated by the activation of the polyol pathway, (2) increased intracellular formation of advanced glycation end-products (AGE), (3) increased expression of the AGE receptor and activation of the ligands, (4) protein kinase C (PKC) isoforms activation, and (5) hyperactivity of the hexosamine pathway. Today, it has been widely accepted that all of these pathogenic mechanisms stem from the HG-induced overproduction of the superoxide anion radical (•O^2−^) in the mitochondrial ETC.

Specifically, high levels of blood glucose increase AGE formation and subsequent OS [21]. AGE accumulation has been related to diabetes as well as its associated complications, although the signaling pathways and underlying mechanisms are not yet clearly defined. AGEs, via their receptors including AGE-R1/OST-48, AGE-R2/80K-H, AGE-R3/galectin-3, lectin-like oxidized LDL receptor 1 (LOX-1), as well as receptor for advanced glycation end products (RAGE) mediate ROS production and increase the intracellular levels of oxygen derivates such as H_2_O_2_, O^2−^, and NO. AGE interaction with its receptor RAGE induces an increase of NADPH oxidase (NOX): NOX1, NOX2, and NOX4 mRNA levels and activates it. In addition, it activates the mitochondrial respiratory chain, microsomal enzymes, and xanthine oxidase and enhances the release of arachidonic acid metabolites [21]. Moreover, AGE-induced ROS upregulates the expression and activities of antioxidant enzymes such as catalase, glutathione peroxidase (GPx), and SOD. AGE-induced increased ROS formation also modulates the signal transduction pathways such as RAS/MEK/extracellular signal-related kinases 1/2 (ERK1/2), phosphoinositide 3-kinase (PI3K)/protein kinase B (Akt), or p38 mitogen-activated protein kinase (MAPK) p21RAS; enhances nuclear factor-kappa B (NF-κB) translocation and activation; and finally induces inflammation, apoptosis, and proliferation by NOD-like receptor protein 3(NLRP-3) inflammasome and the production of several cytokines such as tumor necrosis factor alpha (TNF-α), monocyte chemoattractant protein-1(MCP-1), interleukin-6 (IL-6), and interleukin-1 beta (IL-1β) [21]. Many studies have shown increased circulating levels of IL-6 and TNF-α in rodents and T2DM patients [22]. These cytokines impair insulin signaling and peripheral glucose uptake and contribute to insulin resistance, lipolysis, and hepatic glucose production.

## 3. Astaxanthin: Biological Effect on Diabetes Onset, Progression, and Chronic Complications

### 3.1. General Aspects of Astaxanthin: Structure, Sources, Bioactivity, and Administration Toxicity

#### 3.1.1. Sources and Chemical Structure of Astaxanthin

ASX is one of the most powerful carotenoids with antioxidant activity which is produced naturally or chemically. Natural sources of ASX include various microorganisms and marine animals such as yeast, microalgae, trout, salmon, krill, shrimp, and crayfish; complex plants; and some birds. ASX is not synthesized in the human body but is ingested in the diet, in particular by seafood [23]. Commercialized ASX is often extracted from *Phaffia rhodozyma*, *Blakeslea trispora*, and *Haematococcus pluvialis* as well as is produced by chemical synthesis. *H. pluvialis*, the main source of natural ASX for human consumption, is a unicellular green microalga which accumulates ASX under environmental stress conditions such as starvation, irradiation, high salinity, temperature, or light [24,25]. ASX was authorized in 1987 by the United States Food and Drug Administration (US FDA) as a fish feed additive and then was approved as a dietary supplement in 1999 [26]. ASX has also been registered as a fish feed additive by the European Food Safety Authority (EFSA). According to the Novel Foods Regulation (EU) 2015/2283, ASX from *H. pluvialis* has an acceptable daily intake in human up to 8 mg [27]. The *H. pluvialis*-extracted ASX included in human dietary supplements has been proven to be safe and accepted by the US FDA at daily doses of 2–12 mg [28,29].

ASX (3,3′-dihydroxy-β,β′-carotene-4,4′-dione, molecular formula C_40_H_52_O_4_, molar mass 596.84 g/mol) is a xanthophyll carotenoid which is composed of 40 carbon atoms organized in two oxygenated β-iononetype rings joined by a polyene chain [7,30,31]. The conjugated double bond chain acts as a strong antioxidant by electron donation and by reacting with free radicals [32]. Hydroxyl acid on terminal rings can react with fatty acids and can form monoesters and/or diester. In nature, ASX can also exist conjugated with proteins (e.g., salmon muscle or lobster exoskeleton). Interacting with fatty acids or proteins stabilizes ASX, which in its free form is unstable and particularly susceptible to oxidation; therefore, this form is mainly produced synthetically or from yeast [32]. ASX can be naturally found in stereoisomers, geometric isomers, and free and esterified forms. Moreover, synthetic ASX may have different conformations including chiral ((3S, 3′S) or (3R, 3′R)) or meso forms (3R, 3′S); however, the chiral stereoisomers are the most prevailing forms. In addition, the polyene chain double bond can be organized as two different conformations: cis or trans. Trans-isomers remain the most widespread form due to the thermodynamic instability of cis carotenoids [11].

#### 3.1.2. Administration Doses and Toxicity Studies

Solving ASX stability drawbacks and parenteral administration problems are of crucial importance for the development of ASX-based therapy. In order to encapsulate, deliver, protect, and/or enhance its in vivo stability, several vectors including polymeric systems, lipid-based carriers, and inclusion complex using cyclodextrins have been proposed [33,34]. A polymeric matrix or a coating layer around a particular compound provides a physical barrier, preserves its biological activity, and enhances its physicochemical stability [23,35,36]. Lipid-based carriers based on microemulsion, nanoemulsion, or an association of both referred as solid lipid nanoparticles or nanostructured lipid carriers were shown to stabilize ASX [33,37,38]. A clinical trial revealed the improvement of ASX bioavailability when incorporated into a lipid-based carrier [39].

Nanoemulsions formulated with ASX and α-tocopherol were administered locally to streptozotocin (STZ)-induced diabetic mice wounds; a faster wound closure was seen. The tissue composition in the presence of nanoemulsion was characterized by an abundance of inflammatory cells, fibroblast formation, and collagen syntheses [40]. Cyclodextrins and natural macrocyclic oligosaccharides were also proposed as an encapsulation system of choice to solubilize ASX due to their toroid-shaped structures combining a rigid lipophilic cavity to a hydrophilic outer surface [41,42]. The noncovalent association of the latter with cyclodextrin improves its solubility in water, bioavailability, stability [43], and release [44,45]. The local delivery of cyclodextrin–ASX complexes from biocompatible polyvinyl alcohol-dextran hydrogels was shown to block OS [36]. In addition, in an ischemic rat model, the same systems neutralized excessive ROS and modulated the expression of endogenous antioxidant genes, suggesting that local release of ASX may reduce the damage to tissues by activating endogenous antioxidant pathways [42].

After oral administration, ASX esters are hydrolyzed selectively during absorption within the intestine and are transported through circulation to the tissues [46]. Due to the presence of polar ends in its structure, ASX can be absorbed better than other nonpolar carotenoids such as lycopene and β-carotene [24]. However, its limited solubility in digestive fluids compromises the uptake by intestinal epithelial cells and its final secretion to lymph as chylomicrons [47,48]. The mechanism involved in absorption, metabolic conversion of ASX, and excretion is not completely elucidated. Besides the chemical structure, the absorption and bioavailability of ASX are influenced by a variety of parameters such as age, gender, smoking, fat, food intake, and so on [49]. Various studies have tested dosages of natural ASX ranging from 1–4 mg/day to 100 mg/day of synthetic ASX and reported a nonlinear dose response for plasma concentrations as well as a lower bioavailability of esterified ASX [49].

From a safety perspective, studies on oral administration of natural ASX have not documented negative effects [27]. For instance, oral administration of high doses (up to 1240 mg/kg/day) over a long period of time (90 days) in rats did not exhibit any toxic effect [50]. In addition, in humans, natural ASX ranging from 8 to 45 mg/day over 4 to 12 weeks showed low adverse effects [51]. ASX has an excellent clinical safety, even when compared to the placebo in randomized studies, at low (up to 12 mg) or high (up to 100 mg) doses of daily administration [27]. Nowadays, only natural ASX has been reported as a human dietary supplement [7], and although in vivo preclinical studies on rats tested the actual safe human doses at least 100–800 times, no critical adverse effect was observed. Safety parameters and specific dose activities in humans should therefore be investigated [52].

Since 2010, 34 clinical studies have been reported on the use of ASX [53], from which 22 have been completed until now. ASX has mainly been tested as a dietary supplement alone or in combination with other molecules for safety and pharmacokinetic information or on various pathologies associated to OS (e.g., stroke, infertility, hyperlipidemia, skin aging, and cognitive disfunction). Currently, two studies are recruiting to investigate the potential of antioxidant therapy (ASX combined with lutein, zeaxanthin, vitamin C, vitamin E, zinc, and copper as a daily administered drug) for diabetic retinopathy (unpublished data, clinical trials N° NCT03310359 and N° NCT03702374). The study of antioxidant therapy involving ASX for diabetic retinopathy has been completed; however, the results have not been published yet. Emerging clinical studies using ASX for diabetic complications treatment reflect the medical interest for this antioxidant.

#### 3.1.3. Bioactivity of Astaxanthin

**Figure 1:**
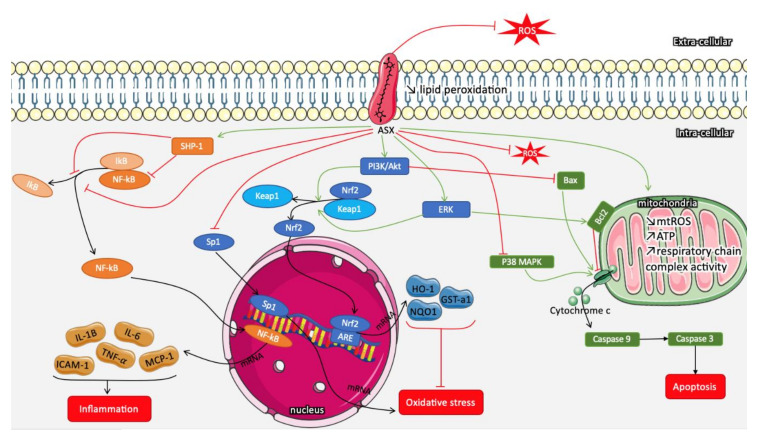
Schematic representation of molecular pathways implied in the protective potential of astaxanthin (ASX): Due to its membrane penetrance, ASX has both intra- and extracellular reactive oxygen species (ROS) scavenging actions. Moreover, in the phospholipid membrane, the ASX polyene chain participates in the reduction of lipid peroxidation. Through the regulation of different pathways, ASX reduces inflammation, oxidative stress, and apoptosis. Red arrows indicate inhibitory action, and green arrows show enhancement action.

At cellular and molecular levels, ASX acts against oxidative damage through various mechanisms (Figure 1) including quenching singlet oxygen, scavenging radicals, inhibiting lipid peroxidation, and regulating OS-related gene expression [54]. ASX’s antioxidant potential is largely attributed to its interactions with cell membrane lipids [55]. In contrast to other carotenoids, such as lycopene and β-carotene, ASX, thanks to its polar structure, is incorporated in the membrane without disorganizing it, decreases lipid hydroperoxide levels, and does not exhibit harmful pro-oxidative effects [56]; in fact, ASX inhibition of lipid peroxidation is related to its ability to trap ROS within and on both sides of the membrane [57,58]. Growing evidence suggests that ASX improves mitochondrial function by reducing mitochondrial reactive oxygen species (mtROS) production and by increasing ATP production, mitochondrial content, and respiratory chain complex activity [59].

Parallel to natural scavenging, in STZ-induced diabetic rats (12 weeks), ASX regulates intracellular OS by mainly activating MAPK, PI3K/Akt, and nuclear factor erythroid 2-related factor 2 (Nrf2)/antioxidant response element (ARE) signaling pathways [60]. Activation of PI3K/Akt and ERK pathways facilitates the dissociation and translocation of Nrf2 to the nucleus. Nrf2/ARE increases the expression of heme oxygenase-1 (HO-1), nicotinamide adenine dinucleotide phosphate (NAD(P)H) quinone dehydrogenase 1 (NQO1), and Glucathione S-transferase alpha1(GST-α1) and leads therefore to an endogenous antioxidant response [52]. ASX was proven to ameliorate early brain injury by the activation of Nrf2/ARE signaling pathways in an experimental subarachnoid hemorrhage rat model [61]. In addition, ASX inhibits the specificity protein 1 (Sp1)/N-Methyl-d-aspartic acid (NMDA) receptor subunit 1 (NR1) pathway, which is involved in intracellular ROS production [54,59].

ASX, through PI3K/Akt and ERK activation, also regulates the Bax/Bcl2 pathway, which reduces cytochrome c release from mitochondria and decreases caspase 3-related apoptosis (reduction of mitochondrial permeabilization). In endothelial cells, under OS, ASX treatment significantly activates PI3K/Akt and reduces the expression of both endothelial nitric oxide synthase (eNOS) and Bax genes [34]. Moreover, ASX decreases caspase-associated apoptosis by modulating the p38 MAPK pathway [62]. Finally, ASX modulates inflammatory response by inhibiting the release of pro-inflammatory cytokines (e.g., interleukin-1B (IL-1B), interleukin-6 (IL-6), intercellular adhesion molecule-1 (ICAM-1), tumor necrosis factor alpha (TNF-α), and monocyte chemoattractant protein-1 (MCP-1)) through the activation of reactive oxygen species (SHP-1), which suppresses NF-κB expression and IκB degradation. ASX also has a direct inhibitory effect on IκB liberation, preventing the nuclear translocation of NF-κB and increasing the anti-inflammatory effect [10,52,63]. The ASX pleiotropic effect was also related to decreased expression of ER markers [64]. For instance, in human liver hepatocellular carcinoma (HepG2), ASX reduces the expression of molecules such as glucose-regulated protein 78 (GRP78), growth arrest and DNA damage-inducible protein (GADD34), C/EBP homologous protein (CHOP), activating transcription factor 4 (ATF4), activating transcription factor 6 (ATF6), and X-box binding protein 1 (XBP1), which are involved in ER stress-induced apoptosis.

### 3.2. Antidiabetic Effects of Astaxanthin

**Figure 2:**
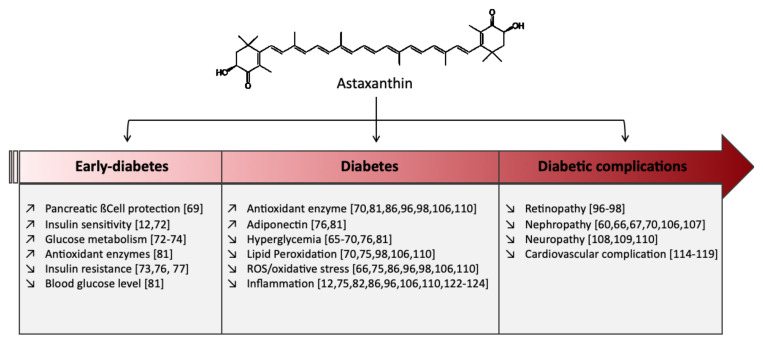
Beneficial effects of astaxanthin at different developmental stages of diabetes mellitus (DM) and its complications.

ASX has shown some beneficial effects on the prevention and treatment of diabetes [6]. These effects depend on how advanced the disease is as well as on the type of complications observed. Figure 2 summarizes the main effects described in the literature. For example, in clinical studies, oral administration of ASX (8 mg/day for 8 weeks) to patients with T2DM significantly reduced the fructosamine and plasma glucose concentrations [65]. ASX also improves glucose metabolism and reduces blood pressure in patients with T2DM.

The antidiabetic effect of ASX has been studied in different animal models with variable outcomes (Figure 2) though the underlying mechanisms have not been clearly elucidated. Oral administration of ASX significantly reduced fasting blood glucose in db/db mice, a well-known obese model for T2DM [66,67,68,69]. A long-term administration of ASX (35 mg/kg for 12 weeks) showed that hypoglycemic effect is linked with a lower malondialdehyde (MDA) content and enhanced SOD activity in serum [70]. In the same animal model, in the presence of ASX (1 mg/day for 18 weeks), pancreatic β-cells were protected against glucose toxicity as they preserved their ability to secrete insulin. The non-fasting blood glucose level was also significantly reduced [69]. Kitahara et al. reported that ASX can also prevent pancreatic β-cell dysfunction by protecting them from ER stress-mediated apoptosis [71]. In pancreatic β-cells mouse insulinoma (MIN6), palmitate-treated, ASX on the one hand reduced MCP-1 and, vascular endothelial growth factor (VEGF) secretion through the, c-Jun N-terminal kinases (JNK) and PI3K/Akt pathways and on the other hand attenuated ER stress by reducing CHOP and by upregulating the expression of GRP78, which is an ER chaperone [71].

Besides the glucose-lowering effect, animal studies have demonstrated that ASX improves insulin sensitivity and glucose uptake, thereby lowering insulin resistance, the hallmark of T2DM. Arunkumar et al. [72] reported that ASX (6 mg/kg per day for 45 days) significantly lowered plasma glucose and insulin levels in HFFD (high fat fructose diet)-fed mice and improved insulin sensitivity. Moreover, ASX potentiates post-receptor insulin signaling events by enhancing the autophosphorylation of insulin receptor-β (IR-β), IRS-1 associated PI3-kinase step, phospho-Akt/Akt ratio, and GLUT-4 translocation in skeletal muscles [72]. Yasuhiro Nishida et al. also showed improved glucose metabolism by enhancing glucose incorporation into skeletal muscles in ASX-treated HFD mice [73]. In this study, ASX administration significantly ameliorated insulin resistance and glucose intolerance through the regulation of 5′ adenosine monophosphate-activated protein kinase (AMPK) activation in the muscle, independent of its antioxidant activity. In addition, it stimulated muscle mitochondrial biogenesis [73].

Furthermore, ASX improves glucose metabolism by increasing glycogen reserves in the liver [74]. It modulates the activity of metabolic enzymes such as hexokinase, pyruvate kinase, glucose-6-phosphatase, fructose-1,6-bisphosphatase, and glycogen phosphorylase. It also promotes the IRS-PI3K-Akt pathway of insulin signaling by decreasing serine phosphorylation of IRS proteins. In STZ-induced diabetic rat, a well-known T1DM model, the treatment with ASX (50 mg/kg body weight/day; 18 days), significantly decreased the levels of AGEs, ROS, and lipid peroxidation in the liver of diabetic rats. These results suggested that the inhibitory effect of ASX on diabetes-induced hepatic dysfunction could be linked to the blocking of AGE formation and further anti-inflammatory effect [75].

Insulin resistance and compensatory hyperinsulinemia are strongly associated with obesity, dyslipidemia, and hypertension in the metabolic syndrome. The effect of ASX was also evaluated on several parameters of metabolic syndrome [6,76]. Hussein et al. reported that a 22-week ASX administration (50 mg/kg/day) in SHRcp (spontaneous hypertensive rat (SHR)/NDmcr-cp (cp/cp)) rats reduced the fasting blood glucose level and improved insulin sensitivity and lipid metabolism parameters including serum adiponectin [76]. Further, it has been shown that ASX administration for 10 weeks prevented and reversed hepatic insulin resistance and ameliorated hepatic steatosis in both genetically (ob/ob) and high-fat-diet-induced obese mice [77]. Moreover, these results were associated with enhanced insulin-stimulated phosphorylation of the insulin receptor (IR)-β subunit (p-IRβ) and Akt (p-Akt) in the livers of CL (cholate)+ASX mice compared with CL mice [77]. ASX may also modulate interactions of peroxisome proliferator-activated receptors gamma (PPARγ) with transcriptional intermediary factor 2 (TIF2), steroid receptor coactivator-1 (SRC-1), 3T3-L1 cell differentiation, and lipid accumulation. PPARγ activation results in changes in adipokine production and in decreased inflammation of adipose tissue, thereby leading to improved insulin sensitivity in the liver and skeletal muscle [12]. Besides PPARγ, ASX activates peroxisome proliferator-activated receptors alpha (PPARα), known to play a role in the regulation of lipid and cholesterol homeostasis [78,79]. ASX diluted in flaxseed oil has been shown to reduce hepatic steatosis as well as hepatic triglycerides and total cholesterol in high-fat diet-fed Sprague–Dawley rats [80]. More specifically, it reduces fatty acid synthesis by increasing PPRAα activity and by lowering lipogenic transcriptional factors and other lipogenic enzymes. Ravi Kumar et al. described the effect of ASX administration combined with squalene in mice fed with high-fat-high-sucrose diet on glucose/triglyceride levels. Blood glucose levels were decreased while adiponectin levels and mRNA expression of Sod1 and GPx were increased, underlining a possible benefit of utilizing the two drugs together for the management of obesity/diabetes [81]. ASX significantly reduces also the expression of ER stress markers (eIF2a (Eukaryotic Initiation Factor 2 alpha), PERK(protein kinase RNA-like endoplasmic reticulum kinase), and Bip (immunoglobulin heavy chain-binding protein)) in high fat fructose fed mice. eIF2a and PERK are implicated in lipogenesis (that also exacerbate ER stress). and Bip stimulates mitochondrial ROS production [82].

Interestingly, recent studies have shown that oral administrated ASX n-octanoic acid diester, a more stable ASX formulation, in high-fat and high-sucrose diet-fed mice improves insulin resistance by modulating gut microbiota [83]. Moreover, it has also been reported that changes in the gut microbiota induced by Xanthophyllomyces dendrorhous-derived ASX administration in high fat fed mice were correlated with changes to some extent in triglyceride and total cholesterol plasma levels [83,84].

On the contrary, some other studies reported no significant influences on glucose/insulin metabolism. For instance, Chan et al. reported no significant hypoglycemic effect of ASX [85]. Preuss et al. examined several parameters of metabolic syndrome in Zucker fatty rats (ZFR); they failed to show an ASX effect on insulin sensitivity [86] at low doses and observed a modulation of glucose-insulin metabolism and nitric oxide at relatively high doses [86]. Differences in administrated doses and/or animal species used among several studies are plausible explanations of the variation in results and stands for a need to further investigate.

### 3.3. Astaxanthin: Protective Effects on Diabetes Complications

OS is a common denominator link for the major pathways involved in the pathogenesis of diabetic micro- as well as macrovascular complications [87]. Hyperglycemia induces the overproduction of mitochondrial superoxide in endothelial cells of both large and small vessels as well as the myocardium. In microvasculature, the intracellular, increased superoxide production causes vascular damage via multiple metabolic pathways including AGE accumulation, AGE receptors, the protein kinase C (PKC) pathway, the polyol pathway, and the hexosamine pathway activation [88]. Inflammation may also play a key role in the development and the progression of diabetes complications [89]. In diabetic macrovascular complications and in the heart, vascular damage appears to be a consequence of increased oxidation of fatty acids, resulting in part in pathway-specific insulin resistance [88].

Owing to its molecular structure, ASX is one of the few antioxidants that can move throughout the body and can provide protection to all of our cells. ASX may help in preventing and treating diabetic microvascular complications by modulating OS, inflammation, and apoptosis through free radical quenching [8]. Although the exact mechanism remains elusive, ASX has been reported to exert beneficial effects in preventing DM complications including retinopathy, nephropathy, neuropathy, and wound healing in numerous diabetic models in animal studies. The recommended dose for reaching positive outcomes with diabetic complications should be addressed in adequate human trials that would evaluate the efficacy and safety of ASX considering the bioavailability, individual response, metabolism, tissue delivery, and possible toxicity.

#### 3.3.1. Astaxanthin: Protection against Diabetic Retinopathy

Astaxanthin has recently been reviewed to have a beneficial pleiotropic effect in the prevention and treatment of several ocular diseases due to its anti-inflammatory, antioxidant, and metabolism regulatory effect [90]. Diabetic retinopathy (DR) is one of the major microvascular complications of diabetes and the most frequent cause of acquired blindness in adults [91]. It is a slow progressive chronic disease, and its prevalence is augmented by the duration of diabetes. The pathophysiology of diabetic retinopathy is complex, as many metabolic changes affect different cell types in the retina. Chronic HG increases OS in the retina and its capillary cells, leading to microvascular damage and retinal dysfunction [92]. HG contributes to nicotinamide adenine dinucleotide phosphate (NADPH) oxidase (NOX) overexpression, leading to cytosolic ROS increase and mitochondrial membrane damage. In retina, OS can activate metabolic pathways including PKC, AGEs, hexosamine pathway, polyol pathway, p38 MAPK pathway, and autophagy pathway can and alter the expression of growth factors such as vascular endothelial growth factor (VEGF) [93]. It can also activate the nuclear factor-κB (NF-κB) transcription factor, leading to the upregulation of pro-inflammatory cytokines that contribute to retinal cell damage and subsequent development of DR [94]. The diabetic environment also compromises the superoxide scavenging system by decreasing the activity of antioxidant enzymes such as SOD, glutathione reductase, glutathione peroxidase, catalase GSH, and manganese superoxide dismutase (MnSOD) activity, as well as by altering enzymes responsible for DNA and histone modifications [92].

**Figure 3:**
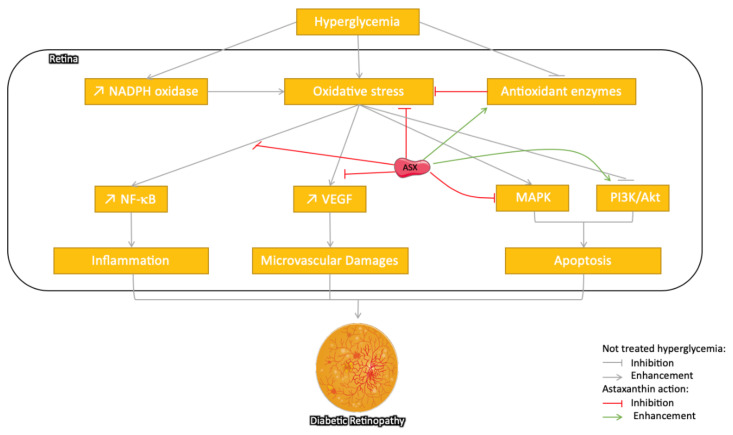
Schematic representation of different pathways suggested for the protective effect of astaxanthin on diabetic retinopathy (DR) via its ROS scavenging potential and reduced oxidative stress induced by the enhanced antioxidant and the downstream pathways activated in hyperglycemic states: In parallel, ASX inhibits inflammation through the nuclear factor-kappa (NF-κB) pathway, microvascular damages through vascular endothelial growth factor (VEGF) production, and finally apoptosis through the regulation of mitogen-activated protein kinase (MAPK) and phosphoinositide 3-kinase/protein kinase B PI3K/Akt pathways. The grey arrow indicates different pathways implied in the development of the pathology, the red arrow indicates the inhibitory/regulatory effect, while the green ones represent a stimulatory effect.

Several studies have reported the protective effect of ASX on DR (Figure 3). In cultured human retinal pigment epithelial cells, ASX reduced the high-glucose exposure-induced increased intracellular level of AGEs, ROS, lipid peroxidation, the mRNA expression of VEGF and matrix metalloproteinase-2 (MMP2), and cell proliferation [95]. In STZ rats, oral administration of ASX plays a protective role in the retinal function and architecture, and so, it prevents DR. ASX reduced the levels of OS mediators (e.g., 8-hydroxy-2′-deoxyguanosine, nitrotyrosine, and acrolein), increased the levels of antioxidant enzymes (heme oxygenase-1 and peroxiredoxin), and decreased inflammatory mediators (intercellular adhesion molecule-1, monocyte chemoattractant protein-1, and fractalkine), which may be mediated by downregulation of the NF-κB transcription factor [96].

An inhibitory effect of ASX has also been recently reported on aldose reductase (AR) activity, a key enzyme in the polyol pathway involved in the pathogenesis of diabetic complications including DR in the gerbil (*Gerbillidae* family, Psammomys obesus species) animal models for T2DM [97]. In db/db mice, ASX improved oscillatory potentials (OPs) and the levels of OS markers including superoxide anion, MDA (a marker of lipid peroxidation), 8-hydroxy-2-deoxyguanosine (8-OHdG, indicator of oxidative DNA damage), and MnSOD (manganese superoxide dismutase) in the retinal tissue [98]. Thus, it was suggested that ASX as an antioxidant possibly improved DR by the regulation of intracellular OS, which mediates pathological cell functionality (VEGF level, inflammation, and excessive cell proliferation).

#### 3.3.2. Astaxanthin Protection against Diabetic Nephropathy

Diabetic nephropathy (DN), also called diabetic kidney disease, is a microvascular complication resulting from lesions in the renal glomeruli and tubuli [99]. It is strongly associated with poor glycemic control [100]. The ROS-related increase of HG induces the recruitment of inflammatory cells [101], the production of inflammatory cytokines [102], and an increased expression of NF-κB [103]. Renal inflammation has been reported to contribute to nephropathy development. ROS overproduction has been also associated with TGF-ß and excessive extracellular matrix (ECM) deposition, leading to the progression of renal fibrosis [104].

**Figure 4:**
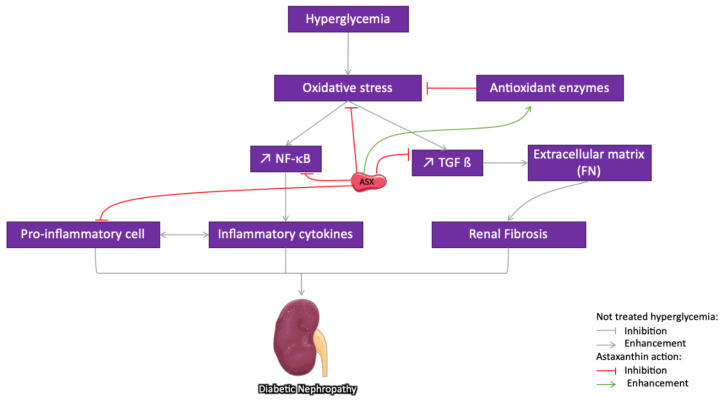
Schematic representation of different pathways suggested for the protective effect of astaxanthin (ASX) on diabetic nephropathy (DN): Through its ROS scavenging and antioxidant potential, ASX reduces oxidative stress and the downstream pathways activated in the development of the disease. In addition, ASX directly inhibits NF-κB translocation and transforming growth factor beta (TGF-β) production as well as reduces inflammation and fibrosis, which are strongly implied in DN. The grey arrow indicates different pathways implied in the DN development, the red arrow indicates an inhibitory/regulatory effect, while the green ones indicate a stimulatory effect.

Several animal studies have reported the nephroprotective effect of ASX and proposed that it should be explored further as a potential remedy for the prevention and treatment of DN (Figure 4). In DN, HG-induced oxidative stress is mainly responsible for the disease progression. Chronic exposure to glucose induces an increase in AGEs that activate the transcription factor NF-κB and the protein kinase C (PKC) system through cellular RAGEs, leading to the inflammation and augmentation of synthesis of the ECM constituents [105]. It has been shown that ASX administration for 12 weeks to db/db mice reversed the HG-induced increased urinary albumin and decreased OS markers such as 8-hydroxydeoxyguanosine [66]. In diabetic rats, dietary supplementation with ASX (20 mg/kg daily) has shown direct favorable effects against renal dysfunction with a return to physiological levels of creatinine and uric acid and a significant reduction in urea level, along with partial reversal of glomerular hypertrophy [67]. ASX increases the activity of antioxidant enzymes such as SOD and catalase and reduces the generated MDA in the serum. It has been also reported that the ASX accumulated in the mitochondria of human mesangial cells suppresses HG-induced ROS production and inhibits the activation of transcription factors and the production of COX-2, MCP-1, and TGFβ1 [106]. ASX may partially interfere with the pathogenesis of DN by reducing the accumulation of ECM components as it regulates the overproduction of fibronectin (FN) and TGF-β1 and can prevent renal fibrosis [60]. Recently, it has been revealed that the renoprotective effect of ASX on DM partially depends on the signaling of the redox-regulated transcription factor Nrf2–ARE that exerts major effects in protecting against oxidative damage [60]. Moreover, in the cultured HG-treated glomerular mesangial cells and in db/db mice, ASX upregulates the Cx43 protein level and inhibits c-Src activity. Furthermore, it promotes the nuclear translocation of Nrf2 and increases the expression level of its downstream protein heme oxygenase-1 and superoxide dismutase 1 [70]. According to a recent report, administration of ASX-s-allyl cysteine biconjugate to SZT-treated rats protected the antioxidant status of kidney and plasma and curbed the progression of secondary complications of DM [107].

#### 3.3.3. Astaxanthin Protection against Diabetes-Induced Neuropathy

**Figure 5:**
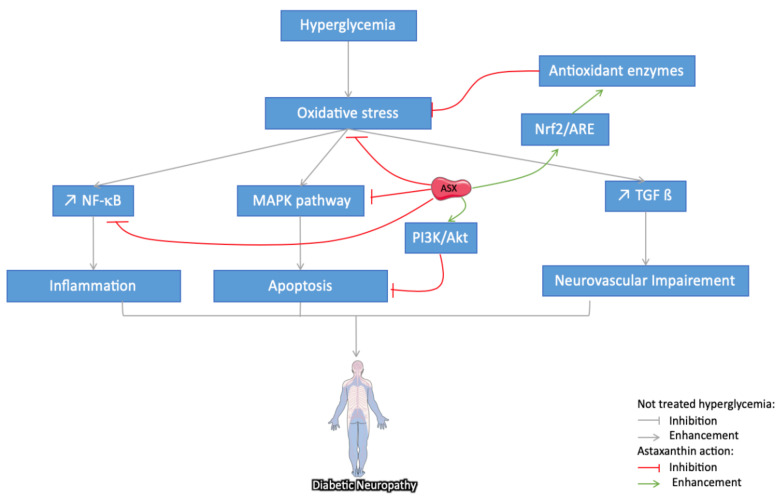
Schematic representation of different pathways suggested for the protective effect of astaxanthin (ASX) on diabetic neuropathy: Through its ROS scavenging potential and the enhancement of antioxidant activities, ASX reduces oxidative stress and the downstream pathways activated in the development of DM-induced neuronal abnormalities. In addition, ASX directly inhibits inflammation through NF-κB, microvascular damages through VEGF production, and finally apoptosis through the regulation of MAPK and PI3K/Akt pathways. The grey arrow indicates different pathways implied in the development of DM-induced neuronal abnormalities, the red arrow indicates an inhibitory/regulatory effect, while the green ones indicate a stimulatory effect.

DM-induced neuropathy and hippocampal-based cognitive deficits are prevalent diseases with important impacts on the life quality of patients. Besides inflammation, the oxidant/antioxidant imbalance is considered the link between the HG states and neuronal abnormalities [6]. Several animal studies have therefore considered the neuroprotective effects of ASX on DM (Figure 5) and on other aging-related diseases, as well [10,52,63]. Specifically, in STZ rats, ASX administration improves cognitive function [108] and simultaneously reduces activities of oxidative stress by increasing the levels of antioxidant enzymes. By reducing NF-κB p65 subunit in the cerebral cortex and hippocampus, ASX reduces the levels of inflammatory mediators such as TNF-α, IL-1β, and IL-6. Furthermore, by activating the PI3K/Akt pathway, it reduces caspase 3 and caspase 9 activities, protecting brain cells from apoptosis. In another study, ASX ameliorated the neuronal behavior in STZ-treated mice. This effect has been related to the decrease of glial fibrillary acidic protein (GFAP) expression in the brain; cleaved caspase-3, IL-6, and IL-1β downregulation; and cystathionine β-synthase (CBS) upregulation in the frontal cortex [109]. In a recent study on diabetic mice, ASX increased the level of SOD and decreased the level of MDA in the hippocampus. It also alleviated cognitive dysfunction by inhibiting OS and inflammatory responses via Nrf2/ARE and NF-κB signaling pathways [110].

#### 3.3.4. Cardiovascular Protective Effect of Astaxanthin in Diabetes

**Figure 6:**
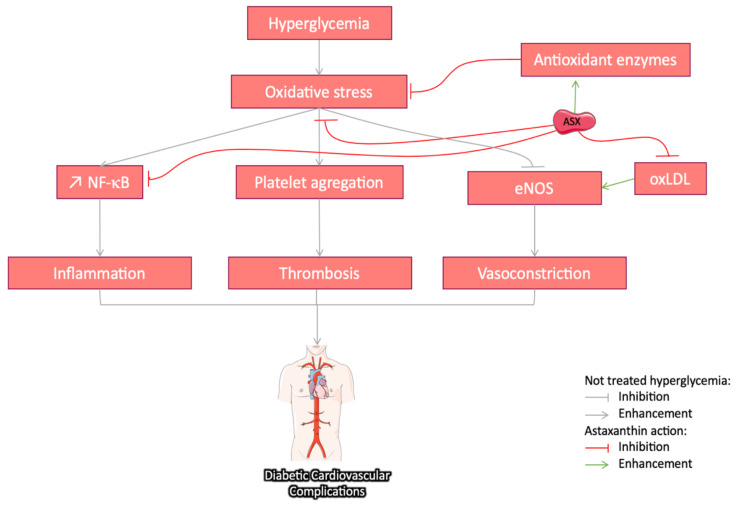
Schematic representation of different pathways suggested for the protective effect of astaxanthin (ASX) on diabetic cardiovascular complications in the literature: through its ROS scavenging potential and the enhancement of antioxidant activities, ASX reduces oxidative stress and the downstream pathways activated in the development of the pathology. In addition, ASX directly inhibits inflammation through NF-κB pathway. Thrombosis and vasoconstriction reduction are also associated with oxidative stress regulation. Moreover, ASX lowers oxidized low-density lipoprotein (oxLDL), enhances endothelial nitric oxide synthase (eNOS), and reduces vasoconstriction. The grey arrow indicates different pathways implied in the development of DM-induced neuronal abnormalities, the red arrow indicates an inhibitory/regulatory effect, while the green ones indicate a stimulatory effect.

Cardiovascular disease is a major cause of mortality in T2DM patients due to hypertensive vascular damages [111]. HG leads to arteriosclerosis through AGE increase [112]. OS resulting from NOX enhancement leads to platelet aggregation and thrombosis. It is worthy to note that, physiologically, the antioxidant enzymes in blood platelets regulate ROS overproduction [113]. Considering that antioxidant enzyme production is compromised by the diabetic environment, ASX could reduce thrombosis through balancing the redox reactions. By limiting the low density lipoprotein, ASX may also contribute to the prevention of arteriosclerosis in a dose-dependent manner [114]. Eventually through the regulation of ROS-induced vasoconstriction, ASX may significantly modulate arterial blood pressure and fluidity in hypertensive animals [115]. The cardiovascular protective effects of ASX and derivatives have been previously reported after prior oral or intravenous administration to different animal species. It also reduces OS and inflammation and is known to contribute to the pathophysiology of atherosclerotic cardiovascular disease [101]. An overview of the potential effects of ASX l (Figure 6) on cardiovascular diseases has been documented in numerous reviews [49,116,117]. It should be noted that DM is one of the contributing events in the cardiovascular physiopathology, besides hypertension, infections of the vessel wall, and smoking. With a focus on the DM context, Zhao et al. studied the effect of ASX (10 mg/kg daily for 42 consecutive days) on endothelial cells in STZ diabetic rats and reported reduced levels of the oxidized low-density lipoprotein (oxLDL) and the expression of its endothelial-cell receptor LOX-1, leading to eNOS expression restoration [118]. Chan et al. showed that, in diabetic rats, dietary intake of astaxanthin (at 0.01 and 0.05%) exhibited anti-inflammatory and anti-coagulatory effect [85]. Moreover, a clinical trial in T2DM patients (daily intake up to 12 mg ASX) significantly attenuated hemostatic disorders and reduced the inflammatory cytokines level without affecting renal function [119]. Further investigations are needed to clarify the mechanisms and the effects of ASX in the DM context.

#### 3.3.5. Anti-Inflammatory Effects of Astaxanthin in Diabetes

Inflammation is a determinant factor in the onset and development of T1DM, T2DM, and their complications [120]. Immune cells involved in pancreatic β-cell destruction are activated through a variety of cytokines including IFN-γ, TNF-α, and IL-1β. Chronic low-level inflammation is a main feature of T2DM, and TNF-α, IL-1, IL-6, IL-10, and adipokines serve as links between inflammation and metabolism. Obese adipose tissue is characterized by macrophage infiltration, which is an important source of inflammation and TNF-α production in this tissue [121].

The anti-inflammatory effects of ASX have been reported in several studies. ASX supplementation induces macrophage phenotype switch by the decrease of pro-inflammatory M1 markers (CD11 and MCP-1) and the increase of anti-inflammatory M2 markers (IL-10) [77,122]. Moreover, Kim et al. [122] observed an attenuated monocyte recruitment, thereby reducing in situ macrophage proliferation. ASX reduces the HG-induced inflammation-related levels of inducible nitric oxide synthase (iNOS), cyclooxygenase (COX), MCP-1, prostaglandin E2 (PGE2), IL-1β, and TNF-α molecules. For instance, in STZ-induced diabetic rats, ASX (0.01% or 0.05% in diet) was shown to reduce the level of activated macrophages (MCP-1) in plasma and kidney and that of the pro-inflammatory cytokines (IL-6 and TNF-α) [85]. Bhuvaneswari et al. demonstrated that, in high-fat-high-fructose diet, ASX administration (2 mg/kg/day) inhibited IkB phosphorylation and therefore the nuclear translocation of the NF-κB p65 subunit [82]. Indeed, numerous studies have demonstrated that the anti-inflammatory effect of ASX is mediated via inhibiting NF-κB activation in diabetes-induced models [123,124]. In fact, by regulating the NF-κB pathway, ASX reduces the risk of complication in diabetes [96,106,108,110]. Thus, ASX in targeting inflammation may improve the prevention and control of the diabetes-related diseases.

## 4. Conclusions and Future Perspectives

OS and inflammation are recognized as determinant factors of DM development and its associated complications. Several in vitro studies have shown a broad range of biological properties in DM-related pathologic cells including pancreatic β-cells, macrophages, T-cells, hepatocytes, adipocytes, endothelial cells, retinal pigment epithelial cells, and glomerular mesangial cells, among others. ASX is able to reduce intracellular oxidative stress and inflammation by scavenging the ROS and inhibition of lipid peroxidation. It modulates multiple OS pathways, but the mechanisms and specificity of action regarding type of cells remain elusive.

The literature reviewed here clearly suggests a promising utilization of ASX in diabetes healthcare. ASX is a safe drug with pleiotropic effects and without crucial adverse effects. The orally/parenterally administrated ASX to T1DM and T2DM animal models has been shown (i) to improve insulin resistance and glucose intolerance, (ii) to reduce hyperglycemia, (iii) to stimulate activation of antioxidant enzymes and reduce OS, and (iv) to have an anti-inflammatory effect. It may have, therefore, a significant preventive role in the onset and progression of DM as well as a protective role in the development of diabetic complications. However, the heterogeneity of DM models, ASX source, dosage, mode of administration, and outcomes studied make comparisons of the obtained results almost impossible.

Although a number of clinical trials have already studied ASX effects, research on the biological mechanisms involved in a diabetic context is necessary. The appropriate dose for reaching positive results with diabetic complications should be addressed in adequate human trials that would evaluate efficacy and safety considering the bioavailability, individual response, metabolism, tissue delivery, and possible toxicity.

## Abbreviations

AGE, advanced glycation end-products; Akt, protein kinase B; ARE, antioxidant response element; ASX, astaxanthin; ATF4, activating transcription factor 4; ATF6, activating transcription factor 4; CHOP, C/EBP homologous protein; COX-2, cyclooxygenase 2; DM, diabetes mellitus; DNA, deoxyribonucleic acid; ER, endoplasmic reticulum; ECM, extracellular matrix; eNOS, endothelial nitric oxide synthase; ERK1/2, extracellular signal-related kinases 1/2; FN, fibronectin; GADD34, growth arrest and DNA damage-inducible protein; GPx, glutathione peroxidase; GRP78, glucose-regulated protein 78; GSH, glutathione; GSTA1, glutathione S-transferase alpha1; HG, hyperglycemia; HO-1, heme oxygenase-1; ICAM-1, intercellular adhesion molecule-1; IκB, inhibitor of nuclear factor kappa B; IL-1, interleukin-1; IL-6, interleukin-6; IR, insulin receptor; JNK, c-Jun N-terminal kinases; LOX-1, lectin-like oxidized LDL receptor 1; MAPK, mitogen-activated protein kinase; MCP-1, monocyte chemoattractant protein-1; MDA, malondialdehyde; MMP2, matrix metalloproteinase-2; MnSOD, manganese superoxide dismutase; mtROS, mitochondrial reactive oxygen species; NADPH, nicotinamide adenine dinucleotide phosphate; NF-κB, nuclear factor-kappa B; NOX, NADPH oxidase; NQO1, NAD(P)H dehydrogenase quinone 1; Nrf2, nuclear factor erythroid 2-related factor 2; oxLDL, oxidized low density lipoprotein; PI3K, phosphoinositide 3-kinase; PKC, protein kinase C; PPAR, peroxisome proliferator-activated receptors; RAGE, receptor for advanced glycation end products; ROS, reactive oxygen species; SHP-1, Src homology region 2 domain-containing phosphatase-1; SOD, superoxide dismutase; SZT, streptozotocin; T1DM, type 1 diabetes mellitus; T2DM, type 2 diabetes mellitus; TGF-ß, transforming growth factor beta; TNF-α, tumor necrosis factor alpha; VEGF, vascular endothelial growth factor; XBP1, X-box binding protein 1.
